# Effect of *T. spiralis* Serine protease inhibitors on TNBS-induced experimental colitis mediated by Macrophages

**DOI:** 10.1038/s41598-020-60155-7

**Published:** 2020-02-21

**Authors:** Jingyun Xu, Lijia Wu, Pengcheng Yu, Yichun Sun, Yixin Lu

**Affiliations:** Heilongjiang Key Laboratory for Animal Disease Control and Pharmaceutical Development, College of Veterinary Medicine, Northeast Agricultural University, 600 Changjiang Street, Harbin, 150030 China

**Keywords:** Autoimmunity, Inflammation

## Abstract

Inflammatory bowel disease (IBD) is an autoimmune disease with increasing incidence rate, and divided into ulcerative colitis (UC) and Crohn’s disease (CD). And more and more experimental evidence supports that immune disorder is important in the pathogenesis of IBD. Our previous experiments have confirmed that TsKaSPI and TsAdSPI recombinant proteins could relieve TNBS (2,4,6-Trinitrobenzenesulfonic acid solution)-induced colitis. Therefore, we speculate that macrophages play a certain role in the process of recombinant protein relieving colitis. In this experiment, 96 male BALB/c mice aged 6–8 weeks were randomly divided into two groups: the prevention group and the therapy group. Changes of the ratio of M1/M2 phenotypic macrophages in spleens and MLNs, key factors in the IL-33/ST2 and IL-6/JAK2/STAT3 signaling pathway were detected. The purpose is to analyze the specific role played by macrophages and their secreted cytokines in the immunomodulation of colitis by *Trichinella spiralis* (*T. spiralis*) Serine protease inhibitors. The results showed that the percentage of M1 phenotypic macrophages was decreased and M2 phenotypic macrophages was increased in the TsKaSPI + TNBS, TsAdSPI + TNBS group compared with the PBS + TNBS group in the prevention group. Meanwhile, the expression of IL-33 and ST2 were significantly decreased. The key factors of IL-6/JAK2/STAT3 signaling pathway were all significantly increased. In addition, in the therapy group, we found similar results. This experiment demonstrated that macrophages have a certain impact during this process of recombinant protein relieving mouse CD model.

## Introduction

The regulation mechanism of *Trichinella spiralis* (*T. spiralis*) on host immune balance is very complicated, immune cells and related cytokines play an important role in this process. SPI (serine protease inhibitor) is currently the most widely studied protease inhibitors superfamily^1^, which plays a vital role in many life activities. Studies have shown that the unique enzyme inhibitory activity of SPI protects the parasite against host digestive digestion, provides favorable conditions for survival in the host, helping the parasite to resist the host immune response^2–5^. Song *et al*. confirmed that vaccination of mice with rTsSPI triggered high level of anti-TsSPI IgG response, and they also demonstrated that TsSPI might participate in the *T. spiralis* larval invasion of IECs and it is likely the potential vaccine target against *T. spiralis* enteral stages^6,7^. Our laboratory has confirmed that TsKaSPI and TsAdSPI are important members of the *T. spiralis* SPI superfamily, which regulate immune responses and play important roles in the inflammatory^8^.

In recent years, the incidence of inflammatory bowel disease (IBD) has increased year by year, and its incidence is closely related to genetic, environmental, microbial, and immune factors, among which the role of immune abnormalities has been widely concerned by scholars^9^. More and more studies have shown that macrophages are involved in the development of IBD, and the number of macrophages is significantly increased in the intestinal mucosa of active IBD patients. In addition, macrophages can also secrete a large number of cytokines and bioactive substances, which are involved in inflammatory responses. Recent experimental studies have confirmed that the conversion between M1 and M2 phenotype could be used as a biomarker to determine whether the body is in the process of inflammatory injury or inflammatory repair^10,11^. During the development of IBD, a variety of factors will break the dynamic balance between M1/M2 phenotype, causing an imbalance in the number and activation, leading to an increasing number of “classically activated” pro-inflammatory M1 macrophages, thus aggravating the inflammatory response^12^. And infect with *T. spiralis* and its derived proteins can lead to the activation of M2 phenotype^13^. Therefore, we speculate that *T. spiralis* and its derived proteins may interfere with the transformation of macrophages from M1 to M2 phenotype, and restore M1/M2 to a balanced state, thereby promoting inflammation regression and tissue repair. So understanding the characteristics of macrophage activation is important for analyzing the mechanism of *T. spiralis* derived proteins in alleviating intestinal inflammation.

Interleukin 33 (IL-33), which transmits signal by binding to the ST2 (growth ST imulation expressed gene 2) receptor, has became a key regulator of a variety of inflammatory diseases, including IBD. After binding to ST2 receptor, IL-33 induces changes in the immune response of the body through signal transduction pathways, and it can also induce the secretion of cytokines such as IL-4, IL-10 and TGF-β^14^, then affect the differentiation and proliferation of Th1, Th2, Treg and other cells. Studies have shown that IL-33 plays a dual immunomodulatory functions of promoting or inhibiting inflammatory reactions in the pathogenesis of IBD^15–17^. However, the latest research showed that IL-33 could induce activation of M2 phenotypic macrophages and promote the secretion of IL-10 and TGF-β by binding to the ST2 receptor on the surface of macrophages, thereby promoting the repair of mucosal epithelial tissue and relieving inflammation, and it was later confirmed that this process was mainly for wound healing^15^. Therefore, this experiment detected the expression of ST2 on the macrophages in MLN (mesenteric lymph node) and IL-33 in colon tissue to verify whether IL-33 and its receptor ST2 play a role in the process of recombinant protein promoting tissue repair of TNBS (2,4,6-Trinitrobenzenesulfonic acid solution)-induced colitis.

JAK2 (Janus kinase 2)/STAT3 (Signal Transducer and Activator of Transcription^3^ pathway is an important signal transduction pathway in the body, which plays a role in various physiological and pathological processes such as immunity, cell proliferation, differentiation, apoptosis and inflammatory response^18^. Under the stimulation of certain cytokines, JAK2 is activated, then activates the STAT3, which can transduce extracellular signals into the nucleus, thereby regulating the expression of related inflammatory factors^19^. Some researchers used gene knockout technology to knock out the mouse STAT3 gene, and found that the pro-inflammatory cytokines secreted by macrophages were significantly increased, suggesting that this signal pathway played an important role in the anti-inflammatory process^20^. In the process of parasitic infection, JAK2/STAT3 signaling pathway also plays an important role. The *Pseudophosphorus* serine protease inhibitor can activate the phosphorylation of the JAK2/STAT3 and induce the conversion of macrophages to M2 phenotype, thus regulating the dynamic balance between pro-inflammatory cytokines and anti-inflammatory cytokines^21^. Therefore, we speculate whether the *T. spiralis* serine protease inhibitor can also activate the phosphorylation of JAK2/STAT3 to alleviate intestinal inflammation.

## Results

### Changes of the expression of M1 and M2 phenotypic macrophages in the spleen and MLN

**Figure 1:**
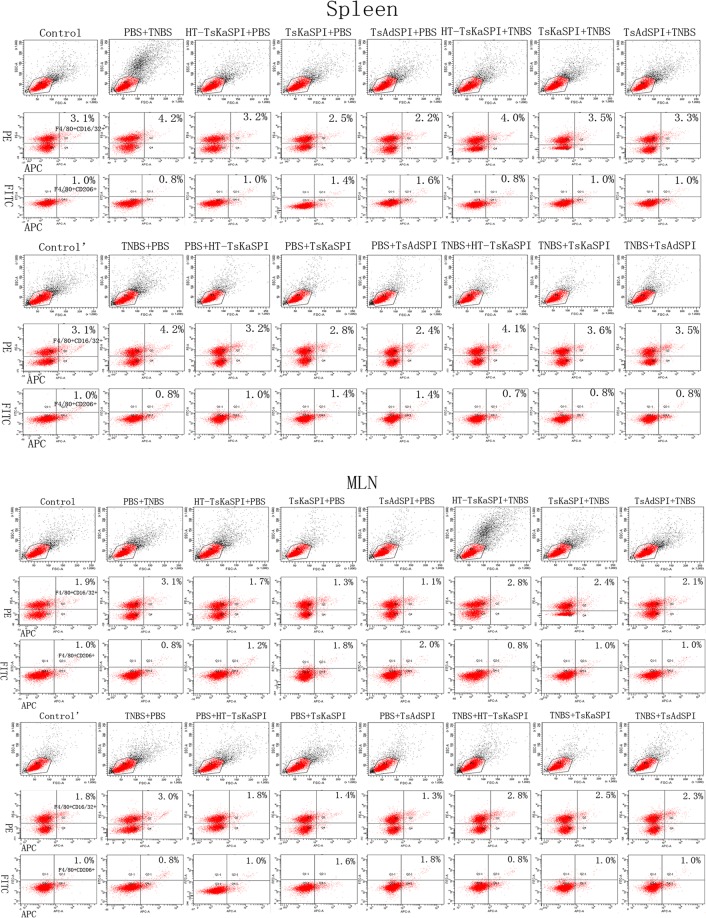
Demonstration of the gating strategy for the flow cytometric analysis of F4/80+CD16/32+ and F4/80+CD206+ phenotypic macrophages in spleens (A) and MLNs (B). In this experiment, single cell suspension were prepared from the spleens and MLNs of each group and stained with F4/80 (APC), CD16/32 (PE), CD206 (FITC) based on staining protocols. Data were collected with FACSDiva flow cytometer and analyzed.

**Figure 2:**
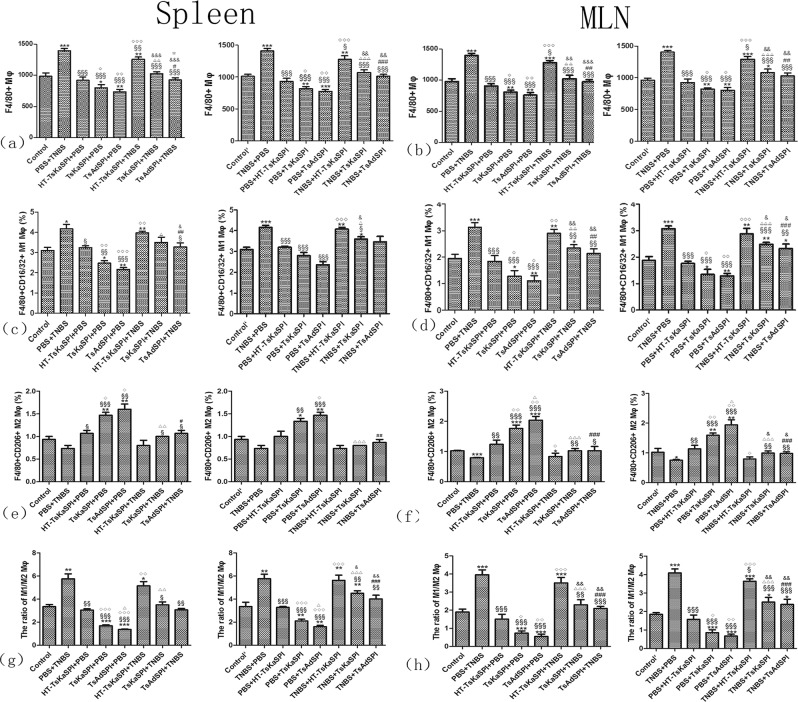
The number of F4/80+ phenotypic macrophages in the 1 × 10^4^ cells in the gate in spleen (**a**) and MLN (**b**), the percentage of F4/80+CD16/32+ phenotypic macrophages in the total number in the Gate in spleen (**c**) and MLN (**d**), the percentage of F4/80+CD206+ phenotypic macrophages in the total number in the Gate in spleen (**e**) and MLN (**f**), the ratio of F4/80+CD16/32+ and F4/80+CD206+ phenotypic macrophages in spleen (**g**) and MLN (**h**) were shown. Data are shown as mean ± SD of 3 mice per group. *P < 0.05, **P < 0.01, ***P < 0.001 versus Control and Control’ group; ^§^*P* < 0.05, ^§§^*P* < 0.01, ^§§§^*P* < 0.001 versus PBS + TNBS and TNBS + PBS group; ^◇^*P* < 0.05, ^◇◇^*P* < 0.01, ^◇◇◇^*P* < 0.001 for HT-TsKaSPI + PBS vs TsKaSPI + PBS^,^ TsAdSPI + PBS, HT-TsKaSPI + TNBS and also for PBS + HT-TsKaSPI vs PBS + TsKaSPI, PBS + TsAdSPI, TNBS + HT-TsKaSPI; ^△^*P* < 0.05, ^△△^*P* < 0.01, ^△△△^*P* < 0.001 for TsKaSPI + PBS vs TsAdSPI + PBS, TsKaSPI + TNBS and also for PBS + TsKaSPI vs PBS + TsAdSPI, TNBS + TsKaSPI group; ^#^*P* < 0.05, ^##^*P* < 0.01, ^###^*P* < 0.001 for TsAdSPI + PBS vs TsAdSPI + TNBS and also for PBS + TsAdSPI vs TNBS + TsAdSPI group; ^&^*P* < 0.05, ^&&^*P* < 0.01, ^&&&^*P* < 0.001 for HT-TsKaSPI + TNBS vs TsKaS*P*I + TNBS, TsAdSPI + TNBS and also for TNBS + HT-TsKaSPI vs TNBS + TsKaSPI, TNBS + TsAdSPI; ^☆^*P* < 0.05, ^☆☆^*P* < 0.01, ^☆^*P* < 0.001 for TsKaSPI + TNBS vs TsAdSPI + TNBS and TNBS + TsKaSPI vs TNBS + TsAdSPI.

Changes of the percentage of M1 and M2 phenotypic macrophages in the spleen (A) and MLN (B) were shown in the Fig. 1. For the number of F4/80+ phenotypic macrophages in the 1 × 10^4^ cells in the gate, that in the PBS + TNBS group (1393.34 ± 35.12) was significantly higher than the Control group (983.34 ± 54.85) (*P* < 0.001), and that in the TsKaSPI + PBS (798.34 ± 52.52) (*P* < 0.05) and TsAdSPI + PBS group (735.00 ± 39.69) (*P* < 0.01) were significantly lower than the Control group, but that in the HT-TsKaSPI + PBS group (918.33 ± 50.08) was not significantly different from the Control group (*P* > 0.05). Meanwhile that in the HT-TsKaSPI + TNBS (1255.00 ± 37.75) (*P* < 0.01), TsKaSPI + TNBS (1023.34 ± 32.53) (*P* < 0.001) and TsAdSPI + TNBS group (925.00 ± 35.00) (*P* < 0.001) were significantly decreased compared with the PBS + TNBS group. The similar result was observed in the therapy group, and in the MLN also observed the similar result (Fig. 2(a)).

In the spleen, Compared with the Control group, the percentage of F4/80+CD16/32+M1 phenotypic macrophages in the PBS + TNBS group (4.17 ± 0.38) was significantly increased (*P* < 0.05), and that in the TsKaSPI + PBS (2.47 ± 0.23) (*P* < 0.05) and TsAdSPI + PBS group (2.17 ± 0.15) (*P* < 0.01) were significantly decreased in the prevention group. Meanwhile, that in the HT-TsKaSPI + TNBS (3.97 ± 0.15), TsKaSPI + TNBS (3.50 ± 0.44) and TsAdSPI + TNBS group (3.27 ± 0.35) were lower than the PBS + TNBS group, but there was only signicant difference between the TsAdSPI + TNBS and PBS + TNBS group (*P* < 0.05). The similar trend was observed in the therapy group, but there was signicant difference between the TNBS + TsKaSPI (3.60 ± 0.17) and TNBS + PBS group (4.17 ± 0.15) (*P* < 0.05). In the MLN, the similar change trend was also observed, and the percentage in each group were all slightly higher than that in the spleen (Fig. 2(b)).

For the percentage of F4/80+CD206+ M2 phenotypic macrophages in the prevention group in the spleen, that in the PBS + TNBS group (0.74 ± 0.12) was lower than the Control group (0.94 ± 0.12), but there was no significant difference. And compared with the Control group, that in the TsKaSPI + PBS (1.46 ± 0.12) and TsAdSPI + PBS group (1.60 ± 0.20) were significantly higher (*P* < 0.01), but there was not significantly difference compared with the HT-TsKaSPI + PBS group (1.07 ± 0.12) (*P* > 0.05). Meanwhile, there was significant difference compared the TsKaSPI + TNBS (1.00 ± 0.06) and TsAdSPI + TNBS group (1.06 ± 0.12) with the PBS + TNBS group (*P* < 0.05), and no significantly difference between the HT-TsKaSPI + TNBS (0.80 ± 0.20) and PBS + TNBS group (*P* > 0.05). In the therapy group, that in the PBS + TsKaSPI (1.34 ± 0.12) and PBS + TsAdSPI group (1.46 ± 0.12) were respectively lower than the TsKaSPI + PBS and TsAdSPI + PBS group, and the percentage in the TNBS + TsKaSPI (0.80 ± 0.04) and TNBS + TsAdSPI group (0.86 ± 0.12) were also respectively lower than the TsKaSPI + TNBS and TsAdSPI + TNBS group. In the MLN, the similar change trend was observed (Fig. 2(c)).

In the prevention group in the spleen, compared with the Control group (3.34 ± 0.32), the ratio of M1/M2 phenotypic macrophages was significantly increased in the PBS + TNBS group (5.75 ± 0.75) (*P* < 0.01), and that in the TsKaSPI + PBS (1.69 ± 0.15) and TsAdSPI + PBS group (1.36 ± 0.08) were significantly decreased (*P* < 0.001). And there was no significant difference compared the TsKaSPI + TNBS (3.50 ± 0.44) and TsAdSPI + TNBS group (3.07 ± 0.21) with the Control group. But that in the TsKaSPI + TNBS and TsAdSPI + TNBS group were significantly lower than PBS + TNBS group, at the same time, the HT-TsKaSPI + TNBS group (5.14 ± 0.63) had not significantly difference compared with the PBS + TNBS group. In the therapy group, the similar trend was observed. In the MLN, there was also observed the similar trend, but the value of the ratio in each group was respectively lower than that in the spleen (Fig. 2(d)).

### Changes of the expression of iNOS and Arg1

In the prevention group, the expression of iNOS in the Control group (9.80 ± 1.19) was significntly lower than the PBS + TNBS group (22.97 ± 0.40) (*P* < 0.001), and significantly higher than TsKaSPI + PBS (5.29 ± 0.84) (*P* < 0.01) and TsAdSPI + PBS group (4.31 ± 0.17) (*P* < 0.001). Meanwhile, that in the TsKaSPI + TNBS (12.38 ± 1.22) and TsAdSPI + TNBS group (10.80 ± 1.41) were significantly decreased compared with the PBS + TNBS group (*P* < 0.001), and there was no significant difference compared with the Control group. The similar trend was found in the therapy group.

**Figure 3:**
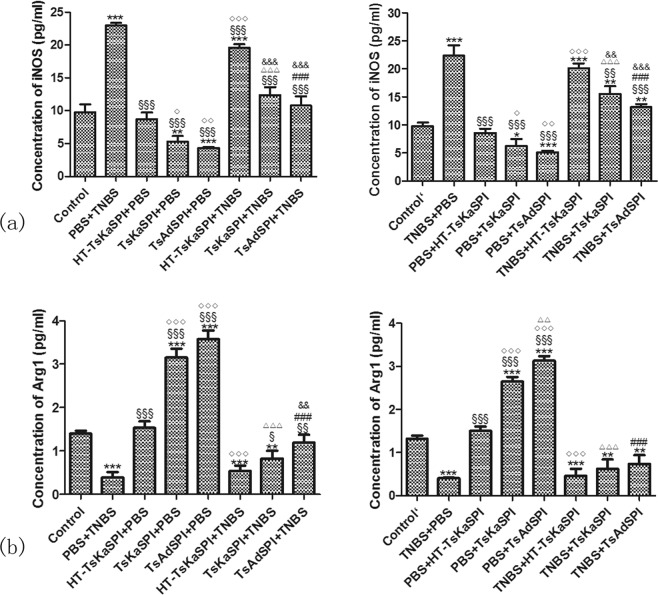
Changes of iNOS and Arg1 in the F4/80+ phenotypic macrophages culture supernatant were tested by using ELISA. Data are shown as mean ± SD of 3 mice per group. The labels representing the significant differences between the different groups are consistent with those in Fig. 3.

The expression of Arg1 in the Control group (1.40 ± 0.07) in the prevention group was significntly higher than the PBS + TNBS (0.39 ± 0.13) (*P* < 0.001), HT-TsKaSPI + TNBS (0.53 ± 0.12) (*P* < 0.001), TsKaSPI + TNBS (0.82 ± 0.18) (*P* < 0.01) and TsAdSPI + TNBS group (3.58 ± 0.20) (*P* > 0.05), and significantly lower than TsKaSPI + PBS (3.15 ± 0.21) and TsAdSPI + PBS group (3.58 ± 0.20) (*P* < 0.001). Meanwhile, that in the TsKaSPI + TNBS (*P* < 0.05) and TsAdSPI + TNBS group (*P* < 0.01) were significantly increased compared with the PBS + TNBS group. The similar trend was found in the therapy group (Fig. 3).

### Changes of the expression of cytokine in F4/80 + phenotypic macrophages culture supernatant

Compared with the Control group, the expression of IL-23 and IL-12 p40 in the PBS + TNBS were significantly increased, and that in the TsKaSPI + PBS and TsAdSPI + PBS group were significantly decreased. And that in the TsKaSPI + TNBS and TsAdSPI + TNBS group were significantly lower than the PBS + TNBS group. However, there was no significantly difference between the HT-TsKaSPI + TNBS and PBS + TNBS group.

**Figure 4:**
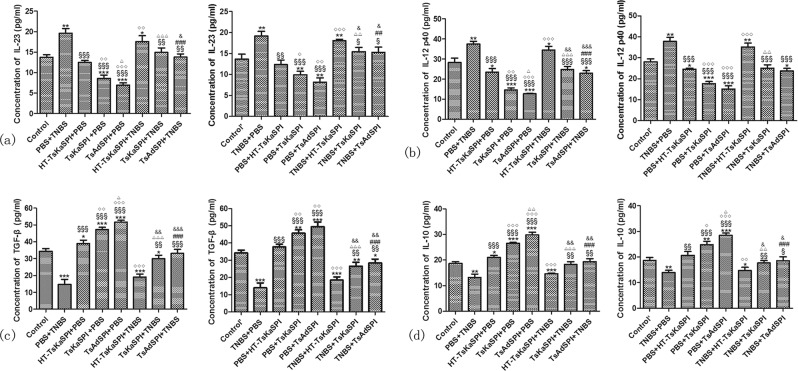
Changes of IL-23, IL-12 p40, TGF-β and IL-12 p40 in the F4/80+ phenotypic macrophages culture supernatant were tested by using ELISA. Data are shown as mean ± SD of 3 mice per group. The labels representing the significant differences between the different groups are consistent with those in Fig. 3.

For TGF-β and IL-10, compared with the Control group, that in the PBS + TNBS were significantly decreased, and that in the TsKaSPI + PBS and TsAdSPI + PBS group were significantly increased. Meanwhile that in the TsKaSPI + TNBS and TsAdSPI + TNBS group were significantly higher than the PBS + TNBS group, and compared the HT-TsKaSPI + TNBS with PBS + TNBS group, there was no significantly difference. The similar trend was observed in the therapy group (Fig. 4).

### Changes of the expression of IL-33 and ST2

**Figure 5:**
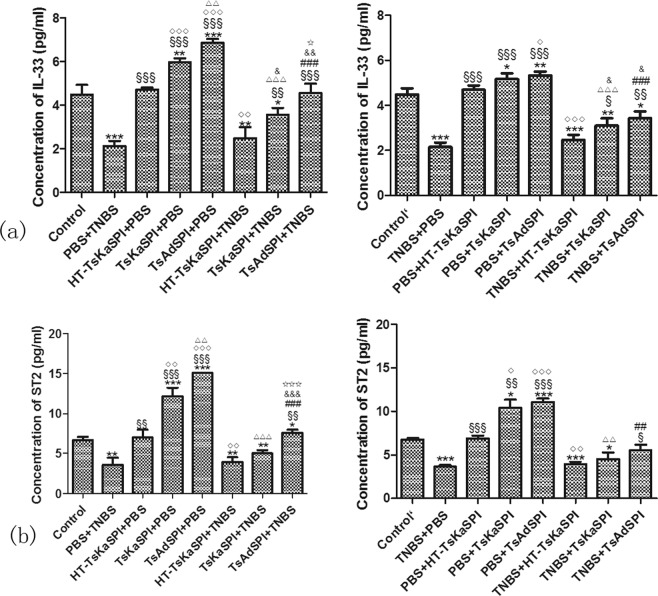
The expression of IL-33 in the colon tissue and ST2 on the F4/80+ phenotypic macrophages were tested by using ELISA. Data are shown as mean ± SD of 3 mice per group. The labels representing the significant differences between the different groups are consistent with those in Fig. 3.

In the prevention group, the expression of IL-33 and ST2 in the Control group were significntly higher than the PBS + TNBS group, and significantly lower than the TsKaSPI + PBS and TsAdSPI + PBS group. Meanwhile, that in the TsKaSPI + TNBS and TsAdSPI + TNBS group were significantly increased compared with the PBS + TNBS group, but there was no significantly difference between the HT-TsKaSPI + TNBS and PBS + TNBS group. In the therapy group, the expression of IL-33 and ST2 in the PBS + TsKaSPI and PBS + TsAdSPI group were lower than that in the TsKaSPI + PBS and TsAdSPI + PBS group. And that in the TNBS + TsKaSPI and TNBS + TsAdSPI group were also lower than that in the TsKaSPI + TNBS and TsAdSPI + TNBS group (Fig. 5).

### Changes in the degree of phosphorylation of JAK2 and STAT3

**Figure 6:**
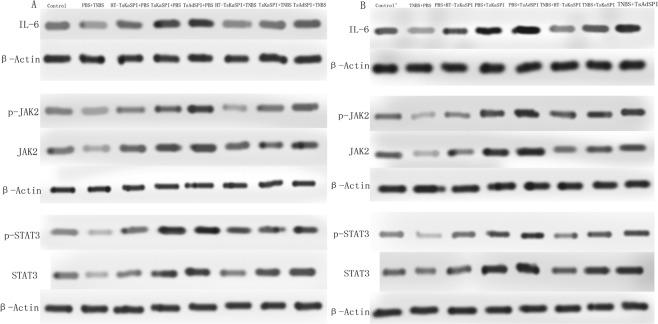
The representative gel of the Western Blot of IL-6, JAK-2, p-JAK2, STAT3, p-STAT3 and β-Actin.

**Figure 7:**
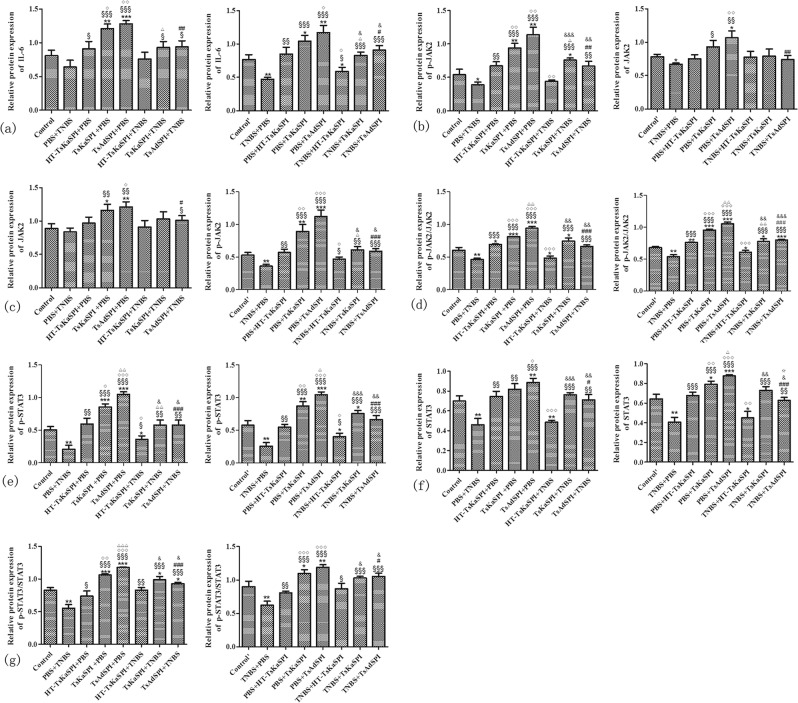
The relative protein expression of IL-6 (**a**), JAK-2 (**b**), p-JAK2 (**c**), STAT3 (**e**), p-STAT3 (**f**) compared with β-Actin and the phosphorylation level of JAK2 (**d**) and STAT3 (**g**) in the colon tissue were showed. Data are shown as mean ± SD of 3 mice per group. The labels representing the significant differences between the different groups are consistent with those in Fig. 3.

The western blotting results of IL-6, JAK2, p-JAK2, STAT3, p-STAT3 were showed in Fig. 6. Compared with the Control group (0.77 ± 0.07) in the prevention group, the relative protein expression of IL-6 in the PBS + TNBS (0.56 ± 0.10) (*P* < 0.05) was significantly lower, while the TsKaSPI + PBS (1.30 ± 0.04) (*P* < 0.01), TsAdSPI + PBS group (1.42 ± 0.10) (*P* < 0.01) were significantly higher, but there was no significantly difference between the HT-TsKaSPI + PBS (0.89 ± 0.09) and control group (*P* > 0.05). Meanwhile, that in the TsKaSPI + TNBS (0.97 ± 0.08) and TsAdSPI + TNBS group (0.98 ± 0.08) were significantly higher than the PBS + TNBS group (*P* < 0.01), and there was no significant difference compared with the Control group. However, compared the HT-TsKaSPI + TNBS (0.70 ± 0.08) with PBS + TNBS group, there was no significantly difference (*P* > 0.05). The similar result was observed in the therapy group (Fig. 7(a)).

In the prevention group, the relative protein expression of p-JAK2 (Fig. 7(b)) and JAK2 (Fig. 7(c)) in the PBS + TNBS group was significantly lower, while the TsKaSPI + PBS and TsAdSPI + PBS group were significantly higher than the Control group. And that in the TsKaSPI + TNBS and TsAdSPI + TNBS group were significantly increased compared with the PBS + TNBS group, there was no significant difference compared the TsKaSPI + TNBS and TsAdSPI + TNBS group with the Control group. However, compared the HT-TsKaSPI + TNBS with PBS + TNBS group, there was no significantly difference. The relative protein expression of p-STAT3 (Fig. 7(e)) and STAT3 (Fig. 7(f)) in the TsKaSPI + PBS, TsAdSPI + PBS group were significantly increased compared with the Control group, and that in the PBS + TNBS group was significantly decreased. While that in the TsKaSPI + TNBS and TsAdSPI + TNBS group were significantly higher than the PBS + TNBS group. However, there was no significantly difference compared the HT-TsKaSPI + TNBS with PBS + TNBS group. The similar result was observed in the therapy group.

The degree of phosphorylation of JAK2 (Fig. 7(d)) and STAT3 (Fig. 7(g)) in the PBS + TNBS group was significantly lower than the Control group in the prevention group, while the TsKaSPI + PBS and TsAdSPI + PBS group were significantly higher, and compared with the PBS + TNBS group, that in the TsKaSPI + TNBS and TsAdSPI + TNBS group were significantly increased. The similar result was observed in the therapy group.

## Discussion

IBD is thought to be the result of the interactions between genes, environment and immune factors, more and more experimental evidence supports that immune disorder is the main factor in the pathogenesis of IBD^22,23^. Macrophages are important immune effector cells in the body and the first line of defense against pathogen invasion, playing an extremely important role in innate immunity and acquired immune response^24^. Macrophages are highly heterogeneous cells that can adapt to different tissue environments and rapidly switch functions^25^. M1 phenotypic macrophages can be identified by the expression of inducile nitric oxide synthase (iNOS) and phenotypic markers such as membrane protein CD16/32, which mainly secrete pro-inflammatory cytokines and chemokines, present antigens, participate in the positive immune response, play the role of immune surveillance, and have the functions of phagocytosis and killing pathogens. M2 phenotypic macrophages can be identified by arginase-1 (Arg-1) expression and phenotypic markers such as CD206, which are produced under the induction of Th2 cytokines such as IL-4, have weak antigen presenting ability, secrete anti-inflammatory cytokines such as IL-10 and TGF-β to anti-inflammatory and promote tissue repair^26,27^. Kayama *et al*.^28^ found that the activation of M1 phenotypic macrophages in peripheral blood and inflammation sites of IBD patients was significantly increased, and the secretion level of inflammatory cytokine was also increased, indicating that the abnormal activation of M1 phenotypic macrophages could promote the inflammatory response of IBD.

However, helminth infection can induce a significant increase in the activation of M2 phenotypic macrophages in the host. M2 phenotypic macrophages down-regulate Th1 type inflammatory response by releasing a large number of anti-inflammatory cytokines, and express resistin-like molecule alpha (RELM-α), Arg-1 and chitinase-3 (Ym1) on inflammatory tissue^29^. Consistent with the effect of helminth infection, the key immunomodulatory molecules of worms can also induce an increase in the activation of M2 phenotypic macrophages in the host^30^. Therefore, we speculate that TsKaSPI and TsAdSPI as members of the *T. spiralis* SPI superfamily may limit the occurrence and development of IBD by inducing the activation of M2 phenotype. Previous experiments have comfired that injected TsKaSPI and TsAdSPI intraperitoneally before or after TNBS-induced mouse colitis model could decreased the levels of inflammatory cytokines and disease activity index (DAI) scores, and significantly reduced inflammation^8^. In this experiment, the number of F4/80+ phenotypic macrophages was significantly decreased, and the percentage of M2 phenotype and the expression level of its effector molecule Arg-1 were significantly increased, the secretion of cytokines IL-10 and TGF-β were increased, and at the same time, the percentage of M1 phenotype and the expression level of effector molecular iNOS were significantly decreased, and the secretion of cytokines IL-12 p40 and IL-23 were reduced, thereby producing a regulation effect on intestinal inflammation.

In recent years, the role of the IL-33/ST2 pathway in IBD has received increasing attention. Seidelin *et al*.^31^ found that the level of IL-33 mRNA in intestinal mucosa of UC patients was significantly higher than that in normal intestinal mucosa, and its expression was significantly correlated with the disease activity level. Behtran *et al*.^32^ detected that the expression levels of ST2 and sST2 mRNA in mucosal tissues of IBD patients were higher than those in the control group, but the expression level of ST2L mRNA in each group were not significantly different. The study by Diaz-Jimenez *et al*.^33^ also confirmed that the expression levels of IL-33 and ST2 were also significantly increased not only in intestinal mucosal tissues, but also in the peripheral blood of UC patients. A large number of studies have confirmed that IL-33 and its receptor ST2 have important clinical significance for UC patients, but for CD patients, larger samples and further studies are still lacking. Since CD mainly presents Th1 type immune response and ST2 receptor is present in Th2 type lymphocytes rather than Th1 type lymphocytes, it is still controversial whether the changes in IL-33 and ST2 expression in CD patients have clinical significance. However, the latest research showed that IL-33 could promote wound healing by binding to the ST2 receptor on the surface of macrophages^16^. Therefore, this study detected the expression levels of ST2 protein on macrophages in MLN and IL-33 in intestinal tissue to analyze whether IL-33 and ST2 proteins were significantly changed in the TNBS-induced CD model, and further judged whether TsKaSPI and TsAdSPI could alleviate intestinal inflammation by affecting the IL-33/ST2 pathway. The results of this experiment showed that the expression levels of IL-33 and ST2 in colitis model animals were significantly lower than those in the Control and Control’ group, and immune proteins could alleviate intestinal inflammation by increasing the expression levels of IL-33 and ST2. Therefore, we can initially confirm that the changes of the IL-33/ST2 pathway in CD model animals are also of great significance.

The IL-6/JAK2/STAT3 signaling pathway is involved in many important biological processes and is closely related to intestinal homeostasis, wound healing response and tumorigenesis^34^, and also plays an important role in the pathogenesis of IBD^35,36^. Kisseleba *et al*.^37^ found that F59D-JAK endogenous inhibitor JAK-binding protein (JAB) transgenic mice showed more severe inflammatory symptoms and higher STAT3 phosphorylation level compared with wild-type mice after induction of colitis by dextran sodium sulfate (DSS). STAT3 is an important member of the cytoplasmic transcription factor family and plays a special role in intestinal immunity of IBD. Driver *et al*.^38^ through animal experiments found that the removal of STAT3 from mouse macrophages and neutrophils led to spontaneous inflammation of the small intestine and colon. Maykel *et al*.^39^ specifically removed STAT3 from mouse bone marrow cells, the mice died 4–6 weeks later and showed intestinal tissue inflammation similar to CD. Similarly, according to our results, the relative protein expression of IL-6, JAK2, p-JAK-2, STAT3 and p-STAT-3 in intestinal tissues of mice in the colitis model group were significantly lower than those in the Control and Control’ group, and the phosphorylation levels of JAK2 and STAT3 were also significantly decreased. Therefore, the activation of IL-6/JAK2/STAT3 pathway is closely related to inhibit the occurrence and development of colitis, which is consistent with the expected results. However, after immunomodulation with TsKaSPI and TsAdSPI, the expression levels of IL-6, JAK2, p-JAK-2, STAT3 and p-STAT-3 proteins were significantly increased, thereby alleviating intestinal inflammation. Therefore, the IL-6/JAK2/STAT3 signaling pathway may be a potential therapeutic target for IBD.

In this experiment, we initially studied that TsKaSPI and TsAdSPI could play a protective and therapeutic role by regulating the activation of macrophages, the expression of key factors in the IL-33/ST2 and IL-6/JAK2/STAT3 signaling pathway in the mouse CD model. Our previous experiments had also confirmed that *T. spiralis* and *T. spiralis* cysteine protease inhibitors had certain therapeutic and protective effects on experimental inflammatory bowel disease induced by TNBS in mice^40,41^, and the effect was not much different from the *T. spiralis* serine protease inhibitors. So whether macrophages also play a role in *T. spiralis* and *T. spiralis* cysteine protease inhibitors relieving colitis remains to be further studied. At the same time, in this experiment, the changes of macrophages were studied preliminary, it remains to be further studied.

## Methods

### Animals

Male Balb/c mice (SPF) aged 6–8 weeks were purchased from the Animal Center of Harbin Medical University. The study protocol was approved by Northeast Agriculture University Veterinary Research Ethics Committee. And all procedures were strictly in accordance with the guidelines of the Chinese National Institute of Health Guide for the Care and Use of Laboratory Animals. Minimized the number of animals used and reduced their suffering. Before the experiment, the mice were allowed to eat and drink freely at least 4 days to adapt to the feeding environment. During the whole experiment, the mice were guaranteed to eat and drink freely, and the bedding materials were changed regularly to ensure the excellent feeding environment.

### Experimental grouping

**Figure 8:**
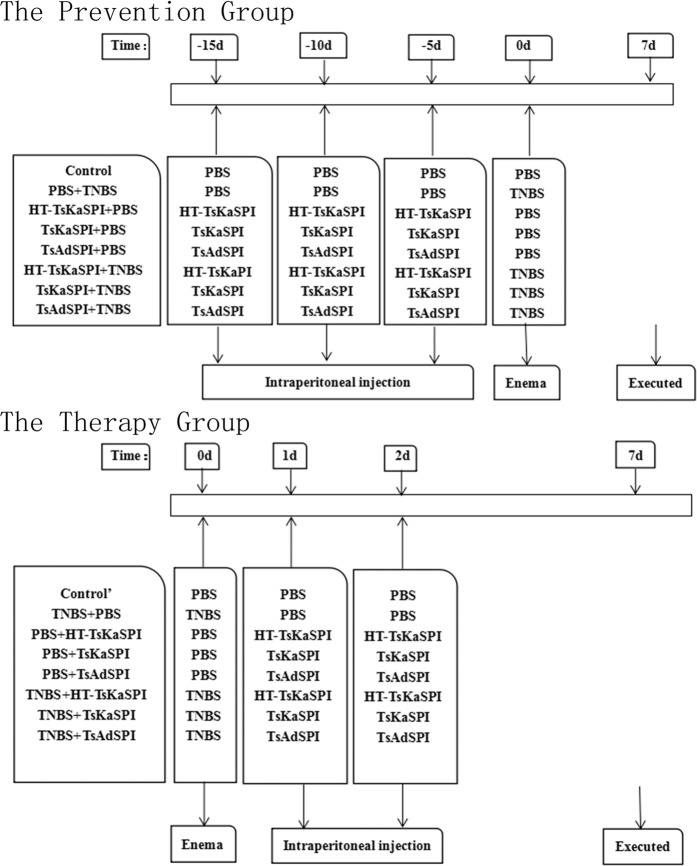
A scheme to clarify the exact moments of immune with protein/PBS and TNBS/PBS administration and the day when the experiment started was day 0.

Preparated the *Trichinella spiralis* Kazal-type serine protease inhibitor (TsKaSPI) and *Trichinella spiralis* adult serine protease inhibitor (TsAdSPI) recombinant protein followed the method described in the previous article^8^. In order to exclude the effect of LPS in the recombinant protein on the experimental results, TsKaSPI was boiled for 10 minutes (HT-TsKaSPI) to denature and inactivate the protein components in it, but LPS was still active, thus serving as a positive control for LPS. The experimental grouping is also consistent with our previous experiment^8^. The experimental mice were randomly divided into two large groups: prevention group and therapy group. The prevention group was divided into eight groups: the Control, HT-TsKaSPI + PBS, TsKaSPI + PBS, TsAdSPI + PBS group: intraperitoneal injection (i.p.) of 50 ug PBS, HT-TsKaSPI, TsKaSPI, TsAdSPI three times at 5d intervals respectively, then intrarectal injection of 100 ul PBS; the PBS + TNBS, HT-TsKaSPI + TNBS, TsKaSPI + TNBS, TsAdSPI + TNBS group: 50 ug PBS, HT-TsKaSPI, TsKaSPI, TsAdSPI three times by i.p. at 5d intervals respectively, then introduction of colitis; And the therapy group was also divided into eight groups: the Control’, PBS + HT-TsKaSPI, PBS + TsKaSPI, PBS + TsAdSPI group: intraperitoneal injection (i.p.) respectively of 50 ug PBS, HT-TsKaSPI, TsKaSPI, TsAdSPI 24 h and 48 h after intrarectal injected with 100 ul PBS; the TNBS + PBS, TNBS + HT-TsKaSPI, TNBS + TsKaSPI, TNBS + TsAdSPI group, 50 ug PBS, HT-TsKaSPI, TsKaSPI, TsAdSPI by i.p. respectively 24 h and 48 h after TNBS administration; six or more mice in each group. All the mice were sacrificed on the seventh day after introduction of colitis and tested for various indexes. The experimental flow chart was shown in Fig. 8.

### Modeling method

TNBS-induced colitis model followed the approach established by Stallmach *et al*.^42^. Mice were anesthetized with 3% sodium pentobarbital after fasting for 24 hours. Then inserted a 1.0 mm thin catheter into the colon, rapidly injected with 100 ul of 2.5 mg TNBS solution (50 ul of 5% (m/v) TNBS in 50 ul of anhydrous ethanol). After injection, pinched the anus and inverted the mice for 3–4 minutes by grabbing the tail. Then restored to normal diet and water. This colitis animal model promotes a Th1-type immune response similar to human CD.

### Flow cytometry (FCM) detection of the expression of M1 and M2 phenotypic macrophages in the spleen and MLN

The spleen and MLN lymphocyte single-cell suspension were prepared^43^ and dispensed into tubes at 1 × 10^6^ cells/tube. The cells were resuspended in PBS and incubated for 30 min in the dark with APC Anti-mouse F4/80, PE Anti-mouse CD16/32 (Sungene Biotech) and FITC Rat Anti-mouse CD206 (Biolegend) at 4 °C. Then washed, and resuspended again for FCM testing. All datas are from three separate experiments.

### Culture of F4/80+ phenotypic macrophages and detection of cytokine

F4/80+ phenotypic macrophages in MLN sorting was performed on the FACSDiva cytometer. After cell sorting, purity was checked (>90%). Macrophages were cultured with RPMI 1640 containing 10% FCS, 2 mM L-glutamine, 0.05 mM β-mercaptoethanol, 100 units/ml penicillin and 100 μg/ml streptomycin in a 37 °C, 5% CO_2_ incubator. The culture supernatant were collected after culturing 18 hours. Added the RIPA Lysis Buffer (biosharp) to the cell sedimentation, and repeatedly sucking several times to dissolve the sedimentation, then centrifuged at 10000–14000 × *g* for 3–5 min to obtain the supernatant, stored at −80 °C until assayed for cytokines. Culture supernatants were assayed for IL-23, IL-12 p40, iNOS, TGF-β, IL-10, Arg1 and the total protein obtained after lysing cells were assayed for ST2 using ELISA kits (Bioss) according to the manufacturer’s instructions. All datas are from three separate experiments.

### Western blotting detection of the expressions of key factors in the JAK2/STAT3 and IL-33 in colon tissues

Performed experiments in strict accordance with Western Blotting operating procedures^44^. Colon tissues were cut and weighed. Added the PIPA Lysis Buffer, and homogenized using a glass homogenizer. After lysis completion, the samples were centrifuged at 10000–14000 × *g* for 3–5 min to obtain the supernatant. Fifty micrograms of colon homogenate proteins were boiled with 5 × SDS-PAGE Sample Loading Buffer and separated by 12% polyacrylamide gel electrophoresis. Proteins were blotted onto a nitrocellulose filter (NC) membrane. The membrane was placed into blocking buffer (5% non-fat milk) for 2 h at room temperature. And the membrane was incubated with the Anti-IL-6, Anti-p-JAK2, Anti-JAK2, Anti-p-STAT3, Anti-STAT3 and Anti-β-Actin (1:5000 diluted in blocking buffer, Bioss) at 4 °C overnight. After being washed using PBST, the membrane was incubated with a peroxidase conjugated secondary antibody, which was diluted in 5% non-fat milk on a shaker for 1 h at room temperature. Being washed, the membrane was exposed using exposure equipment after dropping ultra-sensitive ECL chemiluminescence reagent (Alphabiotech). The bands were quantified by densitometry, and the numbers normalized to β-Actin.

### Statistical analysis

Statistical analysis were performed using EXCEL and SPSS 21. 0. All results were expressed as the mean ± standard error. Data were evaluated using one-way ANOVA analysis. *P* < 0.05 was considered statistically significant. All statistical analysis were performed using GraphPad Prism software.
